# Transcriptome Analysis Revealed Changes of Multiple Genes Involved in *Haliotis discus hannai* Innate Immunity during *Vibrio parahemolyticus* Infection

**DOI:** 10.1371/journal.pone.0153474

**Published:** 2016-04-18

**Authors:** Bo-Hye Nam, Myunghee Jung, Sathiyamoorthy Subramaniyam, Seung-il Yoo, Kesavan Markkandan, Ji-Young Moon, Young-Ok Kim, Dong-Gyun Kim, Cheul Min An, Younhee Shin, Ho-jin Jung, Jun-hyung Park

**Affiliations:** 1 Biotechnology Research Division, Aquaculture Industry Department, National Fisheries Research and Development Institute, Busan 619–902, Republic of Korea; 2 Codes division, Insilicogen Inc., Suwon 441–813, Gyeonggi-do, Republic of Korea; National Cheng Kung University, TAIWAN

## Abstract

Abalone (*Haliotis discus hannai*) is one of the most valuable marine aquatic species in Korea, Japan and China. Tremendous exposure to bacterial infection is common in aquaculture environment, especially by *Vibrio* sp. infections. It’s therefore necessary and urgent to understand the mechanism of *H*. *discus hannai* host defense against *Vibrio parahemolyticus* infection. However studies on its immune system are hindered by the lack of genomic resources. In the present study, we sequenced the transcriptome of control and bacterial challenged *H*. *discus hannai* tissues. Totally, 138 MB of reference transcriptome were obtained from *de novo* assembly of 34 GB clean bases from ten different libraries and annotated with the biological terms (GO and KEGG). A total of 10,575 transcripts exhibiting the differentially expression at least one pair of comparison and the functional annotations highlight genes related to immune response, cell adhesion, immune regulators, redox molecules and mitochondrial coding genes. Mostly, these groups of genes were dominated in hemocytes compared to other tissues. This work is a prerequisite for the identification of those physiological traits controlling *H*. *discus hannai* ability to survive against *Vibrio* infection.

## Introduction

Abalones (Gastropoda; Haliotidae) has become an increasingly important fishery and aquaculture resource with high nutrition value and widely distributed throughout the southern coasts of Korea, Japan, China and Taiwan. Abalones have sedentary lifestyle along shallow rocky coast lines; also these gastropods are vulnerable to capture [1]. Totally, 111,000 metric tons (mt) of abalone (from all sources) were supplied globally in 2013 with over 103,000 mt harvested directly from aquaculture facilities (http://www.fao.org/statistics/en/). China itself account for over 79% of the global abalone aquaculture product followed by South Korea (12%). It is estimated the total output of abalone production in South Korea is more than 1000 mt per year [2].

Like other invertebrates, *Haliotis discus hannai* lacks adaptive immune system and mainly depends on innate immunity. Innate immune system provides a first line for host to defense against invading pathogens and other stress. It is composed of diversified repertoires of receptors, regulators, and/or effectors including Toll-like receptors (TLRs), fibrinogen-related proteins (FREPs), scavenger receptor cysteine-rich (SRCRs), and antimicrobial proteins, as well as many other molecules involved in the key processes of agglutination, phagocytosis, and encapsulation [3]. Immune relevant genes such as ferritin [4], C-type lectin [5], cysteine protease inhibitor [6], cathepsin B [7], chicken-type lysozyme [8] and HSP70 [9] have been separately cloned and characterized from *H*. *discus hannai*. However, knowledge about immune system of this pacific abalone is still fragmentary and different signaling pathways implicated in immune response also remain incomplete.

To date, genome sequence of any *Haliotis* is still unavailable, which limits resources of molecular information. In recent years, *de novo* approach has offered highly-effective technology for analysis of gene expression, discovery of novel transcripts, identification of differentially expressed genes and others [10]. To understand the abalones biology, genomic resources especially transcriptome sequencing for these non-model organisms are necessary. This can lead to the discovery of a large number of candidate genes and address differential gene expression levels that are valuable for further genomic researches.

From broader perspective, earlier studies on the abalone transcriptome is limited but has been increased in recent years [1, 11–14]. Huang et al. [11] used transcriptome sequencing to identify genes that were expressed during early development of abalone. Franchini et al. [1] characterized the South African abalone (*Haliotis midae*) transcriptome with more than 25 million short reads resulting in more than 20,000 relatively short assembled transcripts. Shiel et al. [14] characterized green lip abalone (*Haliotis laevigata*) and mainly focused on the molecular chaperone, Heat Shock Protein 70 (HSP70). Published mitochondrial genomes are available for a few abalone species including *Haliotis rubra* [15] and *Haliotis laveigata* [16] whereas organ specific transcriptomes were studied in *Haliotis asinine* (Jackson et al. 2010) [17] and *Haliotis rufescens* [13]. Organs are connected by substance flow to maintain their overall functions, however the details of which have yet to be made clear at cellular and molecular levels. It has been reported that, hepatopancreas function as an immune organ and primary site to synthesize and excrete immune molecules, such as pattern recognition proteins and lectin or lectin related proteins in all invertebrates [18].

In the present study, we have generated a reference transcriptome for *H*. *discus hannai* that represents multiple tissues responding to multiple stressors common to aquaculture environments. In addition, we have also analyzed hemocytes, gill, hepatopancreas and mantle transcriptome of *H*. *discus hannai* that was infected with the Gram-negative bacteria *Vibrio parahemolyticus*. We further annotated the transcripts by matching them against uniprot, Gene ontology (GO), and Kyoto Encyclopedia of Gene and Genome (KEGG). A subset of these unigenes resulted potential immune molecules of different signaling pathways. The obtained transcriptome data provide an invaluable genetic resource to study the genome and functional genes of abalones and also for comparative genomic studies with other bivalves.

## Materials and Methods

### Abalone and bacterial strains

Abalone, *Haliotis discus hannai* (mean body length 62±1 mm body weight 50±1g, body height 15±1 mm, body width 45±1 mm) were supplied from the Genetics and Breeding Research Center (GRBC) of the National Fisheries Research and Development Institute (NFRDI), Republic of Korea. Abalones were maintained in 6 tons flow-through tank at 18–20°C under a natural photoperiod. The bacterial strain 8M 4–1 which was used for challenge experiment in the present study was isolated originally from the Ark Shell (*Scapharca broughtonii*) during an outbreak of mass mortality in Gangjin bay, Southern Korea in June and September 2012 [19]. According to the analysis of API 20NE profiles and 16S rDNA sequence, the isolate was identified as *Vibrio parahaemolyticus* by API 20NE profiles and 16S rDNA sequence (showing 99% identity with other public reported *V*. *parahaemolyticus*). According to the 50% lethal dose (LD_50_) test with the 8M4-1 strain, the LD50 value was determined at 1.0×10^6^ CFU/mL (data not shown).*V*. *parahemolyticus* from a single working stock was streaked and grown onto a brain-heart infusion (BHI) agar plate (BD Biosciences, USA) at 25°C overnight. To prepare the injection dose, *V*. *paraheamolyticus* was harvested from the plates and suspended in phosphate-buffered saline (PBS), quantified by optical density at 600 nm and then serially diluted in the PBS buffer to obtain the test dosages. The serial diluted bacteria at each OD_600_ values were confirmed by counting colony-forming units (CFU) on the BHI agar plate. OD_600_ values of 0.1 and 1.0 were determined to be equal to approximately 1.2×10^8^ CFU/mL and 1.0×10^9^ CFU/mL, respectively. For validation, abalones were infected with the intracellular injection 100 μL of *V*. *parahemolyticus* (1.2×10^8^ CFU/mL) suspended in PBS buffer, and a control ones were injected with PBS buffer. Tissue samples (hemocytes, mantle, gill and hepatopancreas) from the abalones (three individuals per time point) were collected at 0, 3, 6, 9, and 12 h after infection and immediately ground under liquid nitrogen for RNA extraction. Hemocytes were extracted by centrifugation at 3000rpm for 5min 4°C. In order to check the bacterial challenge, we performed qRT-PCR of *V*. *parahemolyticus* genes (*tlh*, *trh*, *toxR*, *toxRS*) using control and infected samples. Unfortunately, the qRT-PCR results could not obtained significantly due to too short infection time (data not shown). As *H*. *discus hannai* is not an endangered or protected species, and collections were only made from GRBC of NFRDI which is a government funded research institute. Hence, no specific permits were required for the described study.

### Next generation sequencing of transcriptome

To obtain high-throughput transcriptome data of *Haliotis discus hannai*, we implemented Illumina-based NGS sequencing. Total RNA was extracted individually from the six tissues (hemocytes, mantle, gonad, digestive gland, gill and hepatopancreas) of control and four tissues (hemocytes, mantle, gill and hepatopancreas) of infected, using TRIzol reagent (Invitrogen) according to the manufacturer’s protocol. Total RNA was then quantitated using Nanodrop spectrophotometer (Thermo Scientific) and quality-assessed by RNA 6000 Nano assay kit (Agilent) and Bioanalyser2100 (Agilent). NGS sequencing libraries were generated from one microgram of total RNA using TruSeq RNA Sample Prep Kit (Illumina) according to the manufacturer's protocol. In brief, the poly-A containing RNA molecules were purified using poly-T oligo attached magnetic beads. After purification, the total poly A^+^RNA was fragmented into small pieces using divalent cations under elevated temperature. The cleaved mRNA fragments were reverse transcribed into first strand cDNA using random primers. Short fragments were purified with a QiaQuick PCR extraction kit and resolved with EB buffer for end reparation and addition of poly (A). Subsequently, the short fragments were connected with sequencing adapters. Each library was separated by adjoining distinct MID tag. The resulting cDNA libraries were then paired-end sequenced (2x101bp) for control organs with HiSeq™ 2000 system and paired-end sequenced (2x300bp) for infected samples with MiSeq (Illumina).

### Preprocessing, *de novo* assembly and annotations

Paired end sequence files from six tissues (Fastq: R1, R2) were obtained and subjected to processing using Trimmomatic-0.32 with parameter settings like leading:5, trailing:5, sliding window:4:15, and minlen:30. Processed sequences were checked for the bacterial contamination with marine metagenome whole genome shotgun (WGS) sequences (Bio Project: PRJNA13694) downloaded from NCBI. Preprocessed clean reads were mapped to marine metagenome database using Bowtie2 with default parameters and removed those mapped reads with their respective pairs, from now these sequence called as preprocessed. Total, preprocessed sequences from HiSeq were pooled together and assembled with Trinity assembler [20] using default values. To remove the redundant sequences, CD-HIT-EST [21] was used with 95% sequence similarity. To confirm the assembly, the sequence reads from infected samples were mapped to the assembled transcriptome, showing high mapping rate (~ 91%), the MiSeq were not used for the assembly. Finally the transcripts more than ≥500 bp were selected as reference transcriptome. Reference transcriptome were subjected to functional annotation using BLASTX mapping (e-value cut-off 1e^-5^) against to UniProt KB (Metazoa) database and the Gene ontology (GO) terms and Kyoto Encyclopedia of Genes and Genomes (KEGG) pathway maps were using Blast2GO [22]. GO annotations were classified using the WEGO web server [23].

### Identification of differentially expressed genes

Differentially expressed genes (DEGs) were measured by counting tags from infected samples against the non-infected *H*. *discus hannai* and normalized using the RNA Sequence Expected Maximization (RSEM) method [24]. Initially, reads from non-infected tissues (hemocytes, mantle, gill and hepatopancreas) were mapped to reference transcriptome and subjected to check the differential expression using trinity utility scripts (align_and_estimate_abundance.pl and abundance_estimates_to_matrix.pl) as instructed (http://trinityrnaseq.github.io/). From the edgeR statistics files, regulated transcripts across libraries were filtered with default parameters (i.e. 1 ≤ log_2_ (FC), FDR < 0.01) using python scripts. To obtain the differential expression pattern from GenBank datasets, same procedures were followed.

### Experimental validation with qRT-PCR

To validate the transcriptom dataset, five random transcripts from immune-related genes were selected for real-time qPCR confirmation. Primer sequences were designed using (http://fokker.wi.mit.edu/primer3/) and the related information are shown in S4 Table. According to the FastStart DNA Master SYBR Green I (Roche Diagnostics) protocol, the reactions were run on LightCycler system (Roche Diagnostics) using 20 mL reaction system. Reaction procedures were: 95°C 10 s, 45 cycles at 60°C 5 s, 72°C 20 s with fluorescence reading. Immediately following PCR, the machine performed a melting curve analysis by gradually increasing the temperature (0.1°C/s) while measuring the intensity of fluorescence emission. The mRNA expression of each gene was normalized to 18S rRNA expression (accession no. AY319433; 18S rRNA-F, 5’-CTC ACG GAA AGA GCG CGT TTA-3’, 18S rRNA R: 5’-GAC TTG CCC TCC AAT AGA TC-3’) as a reference gene. Each sample was analyzed in triplicate and the data were calculated as the mean ± standard deviation (SD) of relative mRNA expression.

### Sequence information sources

The GenBank database has the most up to date and comprehensive DNA sequence information where scientific communities rely on it for each and everything. To obtain the more precise expression pattern, the sequences were collected from GenBank using the keyword “*Haliotis discus hannai*” and the reference transcriptome were mapped using megablast against to GenBank nucleotide database with the custom parameters (E-value: 100,000, word size: 28, perc_identity:75). Sequences collected from GenBank were mined for the word “Partial/strains” were removed. Mitochondrial genes of *H*. *discus hannai* were obtained from GenBank accession: KF724723 [25]. To confirm completeness of our transcriptome data, reads were mapped with mitochondrial genes using CLC Mapper with default parameters [length fraction 80% and similarity (90%)]. Finally, sequences were selected with ≥ 70% coverage with ≥ 2 depth of individual bases. From here, there sequences named as GenBank datasets (336 genes).

## Results and Discussion

### Transcriptome profile of *H*. *discus hannai*

**Fig 1:**
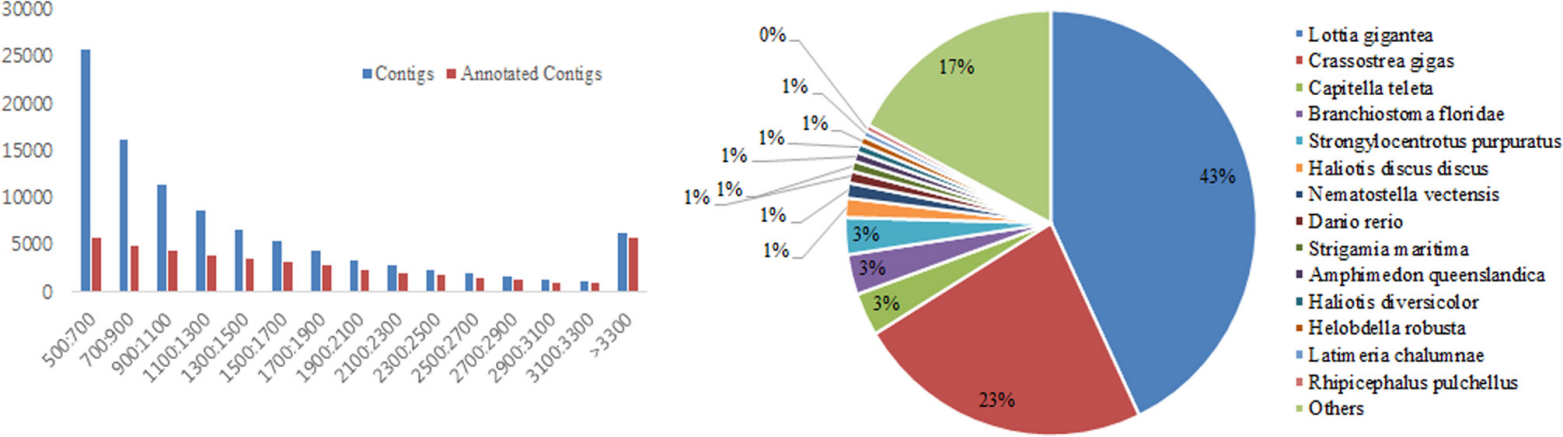
Assembly and annotation summary of *H*. *discus hannai* transcriptome. (A) Comparison of transcript length before and after annotation (B) Species distribution of transcript BLASTX results against the nr protein database. Different colors represent different species. Species with proportions of more than 1% are shown.

To obtain more detailed information of *H*. *discus hannai* transcriptome, adult abalones (6-8cm shell length) were collected and cDNA was transcribed from total RNA followed by cDNA library sequencing with Illumina HiSeq™ 2000 system to obtain as many transcripts as possible. Sequencing of cDNA libraries generated a total of 361,633,550 transcriptomic reads with a length of 36,524,988,550 nucleotides (nt), corresponding to 96% of clean reads. Files containing these data were deposited in the Sequence Read Archive of the National Center for Biotechnology Information (NCBI) with the accession number of SRP059307. Because there is no assembled and annotated *Haliotis* genomic sequences, Trinity *de novo* assembler (Grabherr et al. 2011) was used to assemble all the trimmed reads with optimized Kmer length of 25. The high-quality clean reads were assembled into 97,828 non-redundant unigenes ranging from 500bp to 29,122 bp with an average length of approximately 1,416 bp with a threshold level of ≥500 bp were obtained. The number of unigenes longer than 500 bp is another measure used to compare transcriptome assembly. Among these unigenes, 25,674 unigenes (26.2%) were no more than 700 bp in length, 11,275 unigenes (11.5%) were in the length range of 900 to 1,100bp, and 13,857 unigenes (17.1%) were longer than 2,000 bp. The detailed length distribution of the transcripts is shown in Fig 1A, and a summary of sequencing and assembly results are presented in Table 1.

**Table 1:** Sequencing, assembly and annotations summaries.

***A*. *Sequencing and preprocessing***				
Sampled Conditions	Non Infected		*V*. *parahemolyticus* infected	
No of Samples	6		4	
Sequencing Technology	Hi-seq		Mi-seq	
No of Raw Reads	361,633,550	100%	135,621,610	100%
Total no of Raw Bases	36,524,988,550	100%	36,234,206,749	100%
No of Clean Reads	345,408,880	96%	127,493,803	94%
Total no of clean Bases	34,332,144,915	94%	30,248,043,590	83%
***B*. *Assembly (De novo)***				
No of Transcripts	97,828			
Total Bases	138,588,672			
Length Range (Min-Max)	500 to 29,122			
Mapped Reads by Reference Mapping	117,499,227		91%	
Mapped Bases by Reference Mapping	26,508,379,523		87%	
***C*. *Annotations***				
Blast	43,843		45%	
Gene Ontology	23,560		24%	
KEGG	3,337		3%	

The quality assessment of the transcriptome from *H*.*discus hannai* was evaluated by comparing with other abalone, and the result has been demonstrated that 53% (51,884) of the *H*. *discus hannai* transcripts were similar to the 74% (20,863) unigenes from *H*. *diversicolor* [11] by using blast (1e-3). In addition, according to the each annotation results from other abalones, 23% (8,232) of *H*.*diversicolor* [11], 17% (8,341) of *H*.*midae* [1], 76% (9,968) *of H*. *rufescens* [13, 26], 20% (20,702) of *H*.*laevigata* [14], whereas 45% (43,843) of the *H*. *discus hannai* transcripts were confirmed as having one or more putative annotation. In this results, our study produces more sequencing reads and assembled unigenes.

### Functional annotations

**Fig 2:**
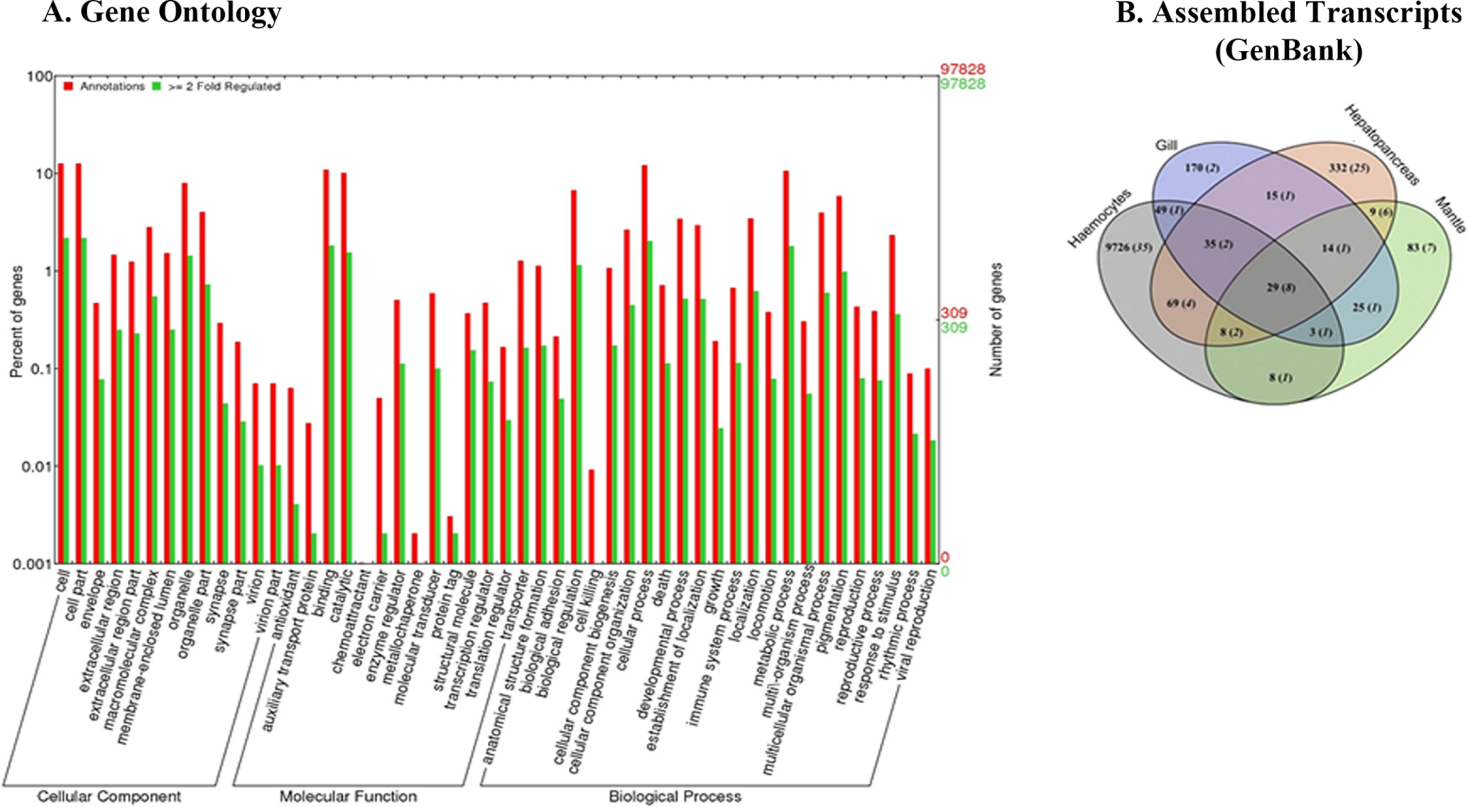
GO categories and 2 fold expressed transcripts. (A) Histogram presentation of GO classification showed transcripts in numbers after annotated in three categories: cellular components, molecular functions, biological processes. (B) Venn diagram shows 2-fold expression of transcripts in *H*. *discus hannai* transcriptome in tissue specific manner. The numbers shown in brackets represent the nucleotide sequences available in GenBank.

To provide putative annotations for the reference transcripts, sequence was annotated using Blast2GO as explained in the method section. A total of 43,843 (45%) unigenes were identified from 97,828 consensus sequences of *H*. *discus hannai*. Among these, 23,560 (24%) unigenes belong to three categories of Gene Ontology (GO), and 3,337 (3%) to 7 categories of KEGG (Table 1). In annotations, the lengthy transcripts were contributed more rather than the short transcripts (Fig 1A) and mostly genes/sequences from *Lottia gigantea* (43%) and *Crassostrea gigas* (24%) were mapped to *H*. *discus hannai* transcripts (Fig 1B). Other bivalve species in the BLASTX top-hit were *Capitella teleta* (3%), *Branchiostoma floridae* (3%) and *Stronglylocentrotus purpuratus* (3%). *H*. *discus hannai* (<1%) itself fell in the least position of the top-hit species distribution. This may be explained by the limited number of *H*. *discus hannai* proteins that currently available at NCBI database. The rest of the low number of matches indicates a lack of bivalve data in public databases. GO analysis of our dataset showed that among 97,828 assembled unigenes, 22,971 of them were successfully annotated by GO assignments to one or more of the three categories: biological process, cellular component and molecular function (Fig 2A), which fell further into 23, 13 and 12 subcategories with the largest ones being the ‘‘cellular process”, ‘‘cell” and ‘‘binding”, respectively.

In summary, these terms account for a large fraction of the overall assignments in the *H*. *discus hannai* transcriptome data. For further identification of the biological pathways in *H*. *discus hannai*, KEGG pathway analysis was performed on all assembled unigenes. Enzyme commission (EC) numbers were assigned to 3,191 unique sequences, which categorized them into 125 pathways. Among those, purine metabolism (2,290), thiamine metabolism and amino benzoate degradation were the top three representatives (S2 Table).

### Differentially expressed genes (DEGs) upon *V*. *parahemolyticus* infection

**Fig 3:**
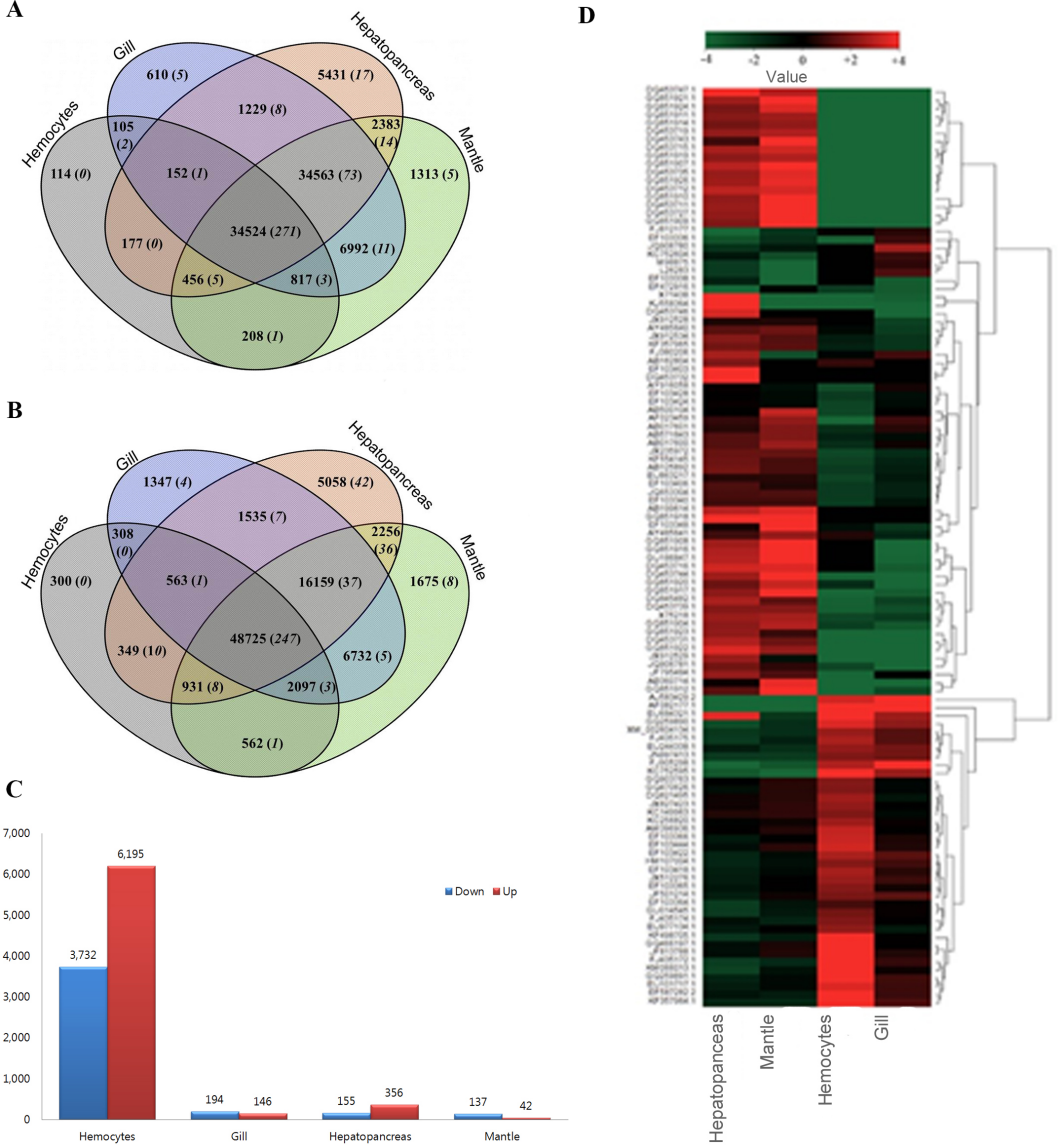
Differentially expressed genes (DEGs) upon *V*. *parahemolyticus* infection. Venn diagrams show DEGs of (A) non-infected and (B) *V*. *parahemolyticus* infected. The numbers shown in brackets correspond to the nucleotide sequences available in GenBank. (C) Tissue specific histogram of up and down regulated transcripts. (D) Heat map of transcripts with expression change more than 2-fold in each tissues.

Crosstalk between stressors and their stress response have been extensively reported in abalones [5, 8, 27–32]. To obtain a general view of DEG expression patterns, pairwise comparison of expression abundance in the RNA-seq data of four library sets were first conducted in accordance with GenBank as described in methods. A Venn diagram in Fig 2B describes, overall, 10,575 transcripts expression were altered in all four tissues after exposure to *V*. *parahemolyticus* infection. Among these tissues hemocytes, hepatopancreas, mantle and gill, a total of 9,726, 332, 83 and 170 transcripts, respectively, were found to be differentially expressed (Fig 3).

Organ/tissue specific expressions were plotted individually for non-infected (Fig 3A) against infected (Fig 3B) *H*. *discus hannai*. The detected fold changes showed that a huge number of transcripts were expressed only in hemocytes (6,195 up-regulated; 3,732 down-regulated) (Fig 3C). In addition, during non-infected condition a significant number of genes were expressed both in hepatopancreas and mantle whereas expression changes observed in both gill and mantle. Further, we checked the full length sequence of our resulted transcripts in GenBank. The results showed that greatest number of genes were up regulated in hepatopancreas and mantle, whereas down regulated in hemocytes and gill (Fig 3D). It has been reported that 78 genes in hemocytes showed differential expression patterns upon bacterial infection in *H*. *diversicolor* [29]. However, most studies in abalones were reported that the tissue specific characterization or expression pattern of genes with respect to dietary supplements [4].

### Validation of *de novo* transcripts using quantitative real-time PCR

In order to validate the transcripts identified from Illumina sequencing, quantitative real-time RT-PCR (qRT-PCR) analysis was performed with randomly selected from immune-related genes (S1 Fig). Results showed that expression value of these genes was in good agreement with the RNA-seq analysis.

### Candidate genes involved in *H*. *discus hannai* immune response

The innate defense mechanisms described in invertebrates have been extensively reviewed [33, 34]. Though there are several reports on isolation and characterization of immune related genes in *H*. *discus hannai* [5–9], molecular basis on the immune response to pathogens are still rare. GO classification revealed 524 and 150 unigenes belongs to two subcategories: response to stimulus and immune system process respectively (Fig 2A). KEGG pathway analysis revealed three signaling pathways, including the T-cell receptor signaling pathway, phosphatidylinositol signaling system and mTOR signaling pathway (S1 Table). These signaling pathways provide comprehensive information for the understanding of the *H*. *discus hannai* immune system. Among them, we focused on key genes corresponding to binding protein, apoptosis and cell cycle related protein, pattern recognition protein (PRR), immune regulator, protease, protease inhibitor, reduction/oxidation (redox) related protein, signal transduction related protein, and stress protein. They were found in our transcriptome dataset infected with *V*. *parahemolyticus*. Subsequently, we selected several genes that have relatively 100% coverage, 2 fold regulated and its full length sequences available in public databases (Table 2).

**Table 2:** Immune response genes (full length; 2 fold) annotated in the transcriptome of *H*. *discus hannai*.

						Log FC			
Putative function	GenBank	No.of. Contigs	Coverage	Species	PMID	Blood	Gill	Hepatopancreas	Mantle
***Apoptosis and cell cycle***									
GTP binding protein Ras^a^	JX512276.1	3	100	*Haliotis discus discus*	NA	2.5	1	-1	-0.1
RAB^a^	EF103422.1	1	100	*Haliotis discus discus*	NA	2.8	1.5	-1.4	-0.4
RAB-1A	EF103367.1	3	99.6	*Haliotis discus discus*	NA	0.8	0.4	-0.8	0
Apoptosis-linked	EF103372.1	3	99.6	*Haliotis discus discus*	NA	0.5	0.3	-0.6	-0.1
DAD1	JX966249.1	2	96.1	*Haliotis diversicolor*	NA	0.5	0.4	-1	-0.7
***Protease and Protease Inhibitor***									
Chymotrypsin-like protease^c^	X71438.1	4	99.2	*H*.*rufescens*	8342947	-7.5	-3.6	4.1	-5.3
Cystatin B^a^	JQ653304.1	43	97.6	*Haliotis discus discus*	22878425	-2.3	-1.2	1.1	1.3
Cysteine proteases inhibitor^b^	JF795484.1	7	99.2	*Haliotis discus hannai*	25463299	-7.1	-0.2	2.3	1.3
Chymotrypsin-like	KJ558364.1	2	97.7	*Haliotis gigantea*	NA	-5.4	-5.9	5	-4.7
Metalloproteinase-1^a^	KM066013.1	2	92.6	*Haliotis rufescens*	25463284	5	0.2	-2.6	-0.7
***Signal transduction***									
C-type lectin	KJ865914.1	1	100	*Haliotis discus hannai*	25301718	0	1.4	0.6	0
Tropomyosin^c^	X75218.1	21	90.1	*H*.*rufescens*	NA	-2.8	-2.4	2.4	2.6
ADP-ribosylation factor 2^a^	EF103418.1	1	100	*Haliotis discus discus*	NA	2.6	0.7	-1.2	-0.3
Calmodulin 2^d^	FJ905298.1	12	99.7	*Haliotis discus discus*	20420919	2.9	5.4	-4.4	-3.3
SOCS-2^a^	EU977134.1	2	99.9	*Haliotis discus discus*	19340953	2.4	-0.9	-1	0
TNF receptor-associated factor	HM581662.1	1	67.5	*Haliotis diversicolor*	NA	4.6	1.5	0	-1.1
paramyosin	AB571843.1	5	100	*Haliotis discus discus*	NA	-1.4	-0.5	1.3	2.1
***Pattern recognition protein***									
Sperm lysin^e^	L26283.1	2	99.9	*Haliotis gigantea*	NA	0	1.3	-2.3	-4.2
TLR^a^	JX827423.1	2	100	*Haliotis discus discus*	23669649	2.3	-0.1	0.3	0.8
PGRP^a^	KF554145.1	9	83.4	*Haliotis discus discus*	24811007	-2.5	-1.3	1.8	1.2
sperm lysin^e^	M98875.1	2	100	*Haliotis discus hannai*	7700151	0	0.8	-2.2	-4.2
***Immune regulator***									
MPEG1	AY485640.1	2	88.7	*Haliotis corrugata*	15020241	-0.4	-2.3	1.5	1.8
MPEG1	KJ558402.1	2	72	*Haliotis discus discus*	24852343	-0.1	-0.2	-0.5	0.3
Rel/NF-kB mRNA	GQ903763.1	1	100	*Haliotis discus discus*	20153832	2.2	-0.2	-0.1	0.7
Allograft inflamatory factor^b^	FJ435175.1	4	99.1	*Haliotis discus discus*	20435145	2.8	1.4	-2.2	-1.3
***Adhesive protein***									
Esterase 1	EF103419.1	1	100	*Haliotis discus discus*	NA	-1.1	-0.4	0.8	1.3
Cyclin B	EF103410.1	3	100	*Haliotis discus discus*	NA	-0.5	0	-0.4	0.7
Nucleoside diphosphate kinase B	EF103393.1	5	100	*Haliotis discus discus*	NA	-0.6	0.2	-0.7	-0.4
Galectin	KJ183034.1	13	100	*Haliotis rufescens*	24952088	0.3	-0.7	0.7	1.3
Transgelin	EF103381.1	5	99.3	*Haliotis discus discus*	NA	0.2	0.8	-0.3	0.1
Gelsolin^a^	EF103444.1	2	100	*Haliotis discus discus*	NA	3.3	0.6	-0.5	0.7
***Redox***									
GST alpha^a^	EF103340.1	9	99.1	*Haliotis discus discus*	NA	-2.3	-0.7	1.1	1.1
GST mu	EF103341.1	11	99.7	*Haliotis discus discus*	NA	0.8	-0.5	-1.6	0
GST omega	EF103342.1	3	96.8	*Haliotis discus discus*	NA	0.8	0.8	0.2	1.1
GST sigma	EF103346.1	1	97.2	*Haliotis discus discus*	NA	-5	-0.8	1.5	0
Glutathione-s-transferase	EF103348.1	2	100	*Haliotis discus discus*	NA	-0.6	-2.1	0.5	4
Ferritin^f^	DQ845482.1	8	100	*Haliotis discus hannai*	NA	-3.8	-2.6	2.7	1.6
Protein disulfide isomerase	EF103409.1	7	100	*Haliotis discus discus*	NA	1.5	0.1	-0.4	0.3
Catalase	DQ821496.1	7	100	*Haliotis discus discus*	18187341	-0.5	-0.9	0.6	0.7
Cu/Zn-superoxide dismutase	DQ530214.1	5	98.7	*Haliotis discus discus*	17574439	-0.3	-0.1	0.1	0
Thioredoxin peroxidase 2	EF103377.1	2	98.7	*Haliotis discus discus*	18226547	0.3	0	-0.7	-0.4
Glutaredoxin 5	EF103397.1	1	97.6	*Haliotis discus discus*	NA	0.7	0.6	-0.8	-0.8
Mn-superoxide dismutase	DQ530210.1	1	97.2	*Haliotis discus discus*	17574439	-0.7	-0.4	-0.1	0.3
***Stress protein***									
HSP90^g^	GU014545.1	8	99.1	*Haliotis discus hannai*	21044885	1.9	0.1	-2.2	-1
HSP26^g^	EF472916.1	6	100	*Haliotis discus hannai*	NA	-2.4	-3.5	-5.1	-0.3
HSP70^g^	FJ812177.1	1	82.4	*Haliotis diversicolor*	NA	0.1	0.7	-5.7	-2.1
***Antimicrobial***									
Histone H2A	EF103400.1	4	99	*Haliotis discus discus*	NA	-0.2	-0.2	-0.1	0.1
Histone H2A isoform 1	FJ380207.1	2	92.4	*Haliotis discus discus*	NA	-0.5	-0.3	-0.3	1
Histone H2A isoform 2	FJ380208.1	5	98.9	*Haliotis discus discus*	NA	-0.8	-0.5	-0.3	0.2
Histone H3	EF103400.1	4	99	*Haliotis discus discus*	NA	-0.2	-0.2	-0.1	0.1
Defensin	DQ520898.1	10	100	*Haliotis discus hannai*	NA	0	0	1.2	-1.5
Defensin	FJ864724.1	2	95.2	*Haliotis discus discus*	19922800	0	0	1.4	0
***Others***									
Alginase^g^	AB199614.1	2	100	*Haliotis discus discus*	NA	0	0	3	3.6
HPRT1	EF103421.1	2	100	*Haliotis discus discus*	NA	1	0.7	-1	0.4
Alginate lyase	JQ353708.1	2	99.9	*Haliotis gigantea*	NA	-4.6	0	2	-0.5
Antistasin-like^e^	FJ380206.1	14	99.7	*Haliotis discus discus*	20060477	-0.3	1.1	2.6	-3.1
Calcineurin A^a^	EF103366.1	10	99.7	*Haliotis discus discus*	NA	3.3	0.5	-0.9	0
Caspase 8	FJ864721.1	10	95	*Haliotis discus discus*	NA	-1.5	-1.6	0.8	1.1
Temptin	EF103375.1	2	99.6	*Haliotis discus discus*	NA	0	0	-0.5	0
Serpin-like^a^	JF813788.1	3	73.3	*Haliotis discus hannai*	NA	4.8	0.5	-0.6	0.5
Calponin	EF542809.1	1	70.6	*Haliotis diversicolor*	18538840	0	0.1	-0.6	-0.5

^a^ Blood

^b^ Blood & Hepatopancreas

^c^ Blood, Hepatopancreas & Mantle

^d^ Blood, Gill, Hepatopancreas & Mantle

^e^ Hepatopancreas & Mantle

^f^ Blood Gill Hepatopancreas

^g^-Hepatopancreas.

C-type lectins (CTLs) play an important role in the innate immunity of invertebrates. It specifically binds to the carbohydrate residues present on the surface of pathogens. CTLs are widely distributed in the marine populations as the first line of defense and well characterized in oysters [35], but less information is available for abalones. Recently, Zhang et al. [5] reported that, a novel C-type lectin from *H*. *discus hannai* (HdCTL1) was involved in host defense against gram-negative bacterial pathogens in the presence of mannose.

Pattern recognition receptors (PRRs) recognize the invading pathogens by innate pattern recognition, and such recognition in the early stage of infection is an important process in the prevention of disease outbreak. Toll-like receptors (TLRs) and peptidoglycan recognition protein (PGRP) are one among the PRRs family, known to induce immune responses against the pathogens by interacting with evolutionarily conserved pathogen-associated molecular patterns (PAMPs) including lipopolysaccharides or peptidoglycan in bacterial cell wall, β-1,3-glucan on fungal cell wall and dsRNA from viruses [36]. These pattern recognition receptors, AbTLR and AbPGRP have been characterized in *H*. *discus discus* [31]. The AbPGRP showed significant homology with other molluscan PGRPs implies that it showed high level of expression in hemocytes. Similar expression pattern of 26 TLR and/or PGRP transcripts were observed in our results during exposure to bacteria. In addition, 494 T cell receptor signaling pathway transcripts were identified in which 53 transcripts showed higher expression (RPKM>0.3) in hemocytes compared to other tissues. Information about transcripts that showed homology to molecules involved in T cell receptor signaling pathway is included in S2 Table.

The engulfment of bacteria by phagocytic cells results in the activation of numerous innate immune signaling pathways. Reactive oxygen species (ROS) generation is one of the most ancient and efficient means to kill pathogens and it is functionally linked to phagocytosis. Free oxygen radicals and antioxidants are highly toxic and prevent colonization of microbial invaders. Glutathione-S-transferase (GST), metal dependent superoxide dismutase (SOD), peroxiredoxin and catalase are the major antioxidants responsible for abalone innate immunity. Previously, a number of redox genes were characterized and their expression pattern was studied widely in abalones [37–44]. It has been reported that almost all these redox genes were expressed only in hemocytes. In our data, large number transcripts were shown to be down regulated in hemocytes, indicate that ROS has been maintain during the bacterial infection (S3 Table).

### In-house analysis with mitochondrial genome

**Fig 4:**
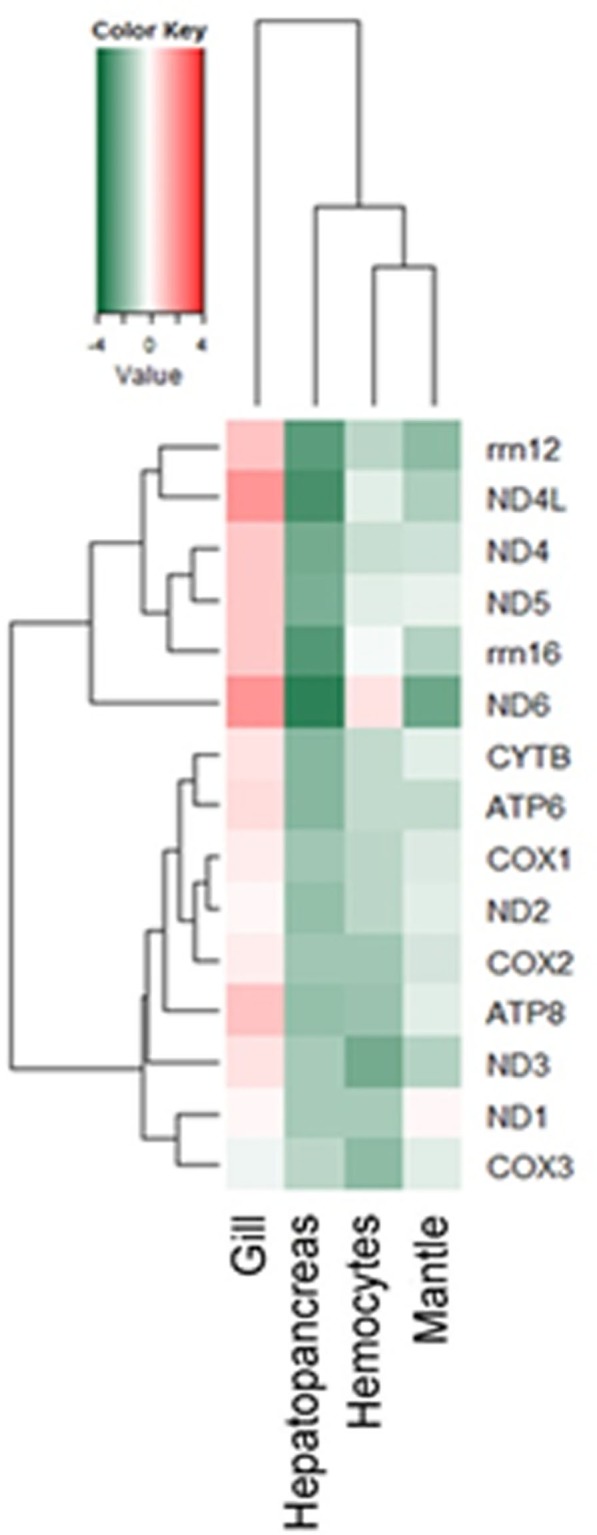
Heat map of mitochondrial genes expressed (2-fold) in *H*. *discus hannai* transcriptome.

Mitochondria emerge as a key component in host innate immune defense, as this organelle is a major cellular source of ROS, which contribute in many ways to host defense against pathogens. We performed an in-house coverage analysis of our infected transcriptome profile with the complete *H*. *discus hannai* mitochondrial genome published recently by our group [25]. The results are presented as total number of reads matching with the genes. Overall, 96.57% (16,307 bp) of the mitochondrial genome was mapped with our transcriptome data. Amongst, 13 coding genes, 2 rRNAs and 3 tRNAs showed 100% coverage with the reads. In contrast, the tRNAs varied enormously in their coverage levels. However, when comparing normalized expression, the most highly expressed genes were nd4L and nd6, especially in gill. Five genes had low expression in hepatopancreas, namely nd4, nd4L, nd6, rrn12 and rrn16. The genes encoding subunits of the respiratory complexes (nd1, nd2, nd3, cytb, cox1 to cox3, atp6 and atp8) showed low expression in all tissues and after normalization (Fig 4).

## Conclusion

This is the first comprehensive investigation on the transcriptome of *H*. *discus hannai*. In this study, comparative analysis of transcriptome changes between *V*. *parahemolyticus* infected and non-infected *H*. *discus hannai* revealed a large number of differentially expressed candidate genes and pathways related to immune response were identified in the transcriptome dataset, providing abundant genomic data for future studies on the molecular mechanisms behind important abalone aquatic environment infected with various pathogens.

## Supporting Information

S1 Fig Immune-related transcripts expression patterns using qRT-PCR. (TIF)

S1 Table KEGG pathways classification from the annotated transcripts. (DOCX)

S2 Table Tissue/organ specific expression of transcripts. (XLSX)

S3 Table Representative of known full length genes from GenBank. (XLSX)

S4 Table The information of qRT-PCR primers. (XLSX)
