# Supplementary figures and images for: Transcriptome Analysis Revealed Changes of Multiple Genes Involved in Haliotis discus hannai Innate Immunity during Vibrio parahemolyticus Infection
Source: PLoS One. 2016 Apr 18;11(4):e0153474. doi: 10.1371/journal.pone.0153474 (PMC4835058; doi:10.1371/journal.pone.0153474)

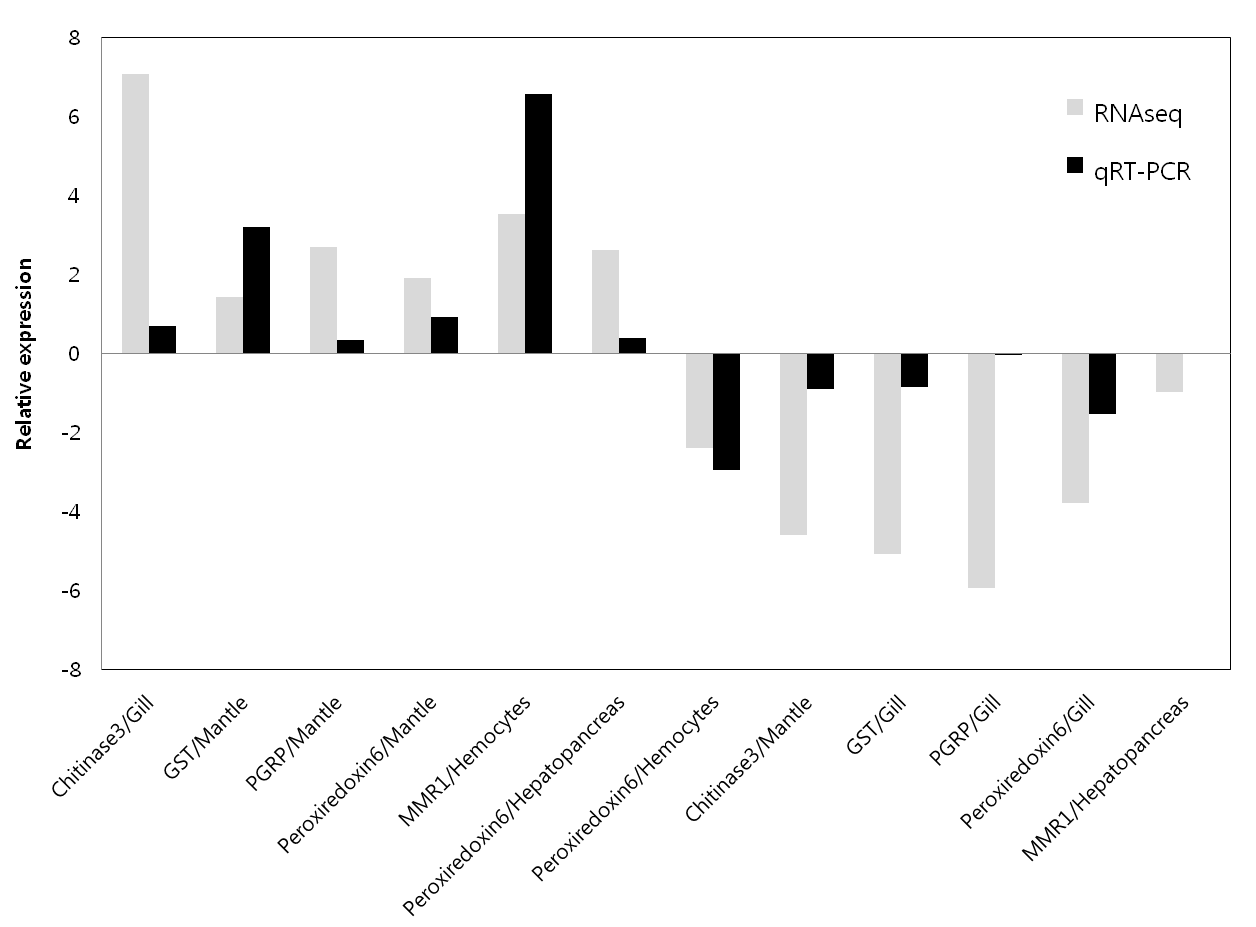

Supplement: S1 Fig — (TIF) [file pone.0153474.s001.tif]
