# Supplementary material for: Transcriptome Analysis Revealed Changes of Multiple Genes Involved in Haliotis discus hannai Innate Immunity during Vibrio parahemolyticus Infection
Source: PLoS One. 2016 Apr 18;11(4):e0153474. doi: 10.1371/journal.pone.0153474 (PMC4835058; doi:10.1371/journal.pone.0153474)
Supplement: S1 Table — (DOCX) [file pone.0153474.s002.docx]

Supplementary Table 1: KEGG pathways classification from the annotated transcripts

| Pathways | Annotated Transcripts | ≥log 2 Fold  Expressed |
| --- | --- | --- |
| Purine metabolism | 2,290 | 185 |
| Thiamine metabolism | 1,746 | 141 |
| Aminobenzoate degradation | 777 | 77 |
| T cell receptor signaling pathway | 494 | 53 |
| Pyrimidine metabolism | 357 | 28 |
| Drug metabolism - other enzymes | 314 | 25 |
| Lysine degradation | 186 | 17 |
| Glycerophospholipid metabolism | 183 | 18 |
| Glycolysis / Gluconeogenesis | 182 | 22 |
| Arginine and proline metabolism | 182 | 19 |
| Tryptophan metabolism | 166 | 8 |
| Starch and sucrose metabolism | 163 | 17 |
| Fructose and mannose metabolism | 163 | 13 |
| Alanine, aspartate and glutamate metabolism | 158 | 17 |
| Amino sugar and nucleotide sugar metabolism | 156 | 18 |
| Pyruvate metabolism | 151 | 18 |
| Glycine, serine and threonine metabolism | 150 | 15 |
| Other glycan degradation | 145 | 17 |
| Phosphatidylinositol signaling system | 144 | 21 |
| Glycerolipid metabolism | 143 | 14 |
| Aminoacyl-tRNA biosynthesis | 138 | 21 |
| Cysteine and methionine metabolism | 138 | 19 |
| Glutathione metabolism | 138 | 9 |
| Valine, leucine and isoleucine degradation | 134 | 11 |
| Carbon fixation pathways in prokaryotes | 133 | 17 |
| Phenylalanine metabolism | 121 | 10 |
| Citrate cycle (TCA cycle) | 117 | 9 |
| Sphingolipid metabolism | 113 | 9 |
| Nicotinate and nicotinamide metabolism | 111 | 6 |
| Pentose and glucuronate interconversions | 107 | 10 |
| beta-Alanine metabolism | 105 | 8 |
| Oxidative phosphorylation | 104 | 10 |
| Fatty acid degradation | 98 | 8 |
| Glyoxylate and dicarboxylate metabolism | 98 | 3 |
| Galactose metabolism | 95 | 15 |
| Inositol phosphate metabolism | 87 | 12 |
| Pentose phosphate pathway | 86 | 8 |
| Butanoate metabolism | 81 | 4 |
| Metabolism of xenobiotics by cytochrome P450 | 80 | 6 |
| Drug metabolism - cytochrome P450 | 77 | 6 |
| Tyrosine metabolism | 75 | 6 |
| Methane metabolism | 74 | 6 |
| Nitrogen metabolism | 72 | 9 |
| Ascorbate and aldarate metabolism | 72 | 8 |
| alpha-Linolenic acid metabolism | 71 | 7 |
| Phenylpropanoid biosynthesis | 71 | 4 |
| Mucin type O-Glycan biosynthesis | 69 | 4 |
| Ether lipid metabolism | 66 | 10 |
| Propanoate metabolism | 65 | 4 |
| Carbon fixation in photosynthetic organisms | 62 | 3 |
| Histidine metabolism | 61 | 4 |
| Arachidonic acid metabolism | 60 | 6 |
| Porphyrin and chlorophyll metabolism | 59 | 14 |
| Pantothenate and CoA biosynthesis | 56 | 2 |
| Limonene and pinene degradation | 55 | 6 |
| One carbon pool by folate | 53 | 12 |
| Selenocompound metabolism | 52 | 6 |
| Chloroalkane and chloroalkene degradation | 51 | 4 |
| Retinol metabolism | 46 | 4 |
| Biosynthesis of unsaturated fatty acids | 45 | 3 |
| N-Glycan biosynthesis | 44 | 13 |
| Glycosaminoglycan degradation | 44 | 4 |
| Fatty acid elongation | 40 | 3 |
| Streptomycin biosynthesis | 39 | 8 |
| Linoleic acid metabolism | 39 | 5 |
| mTOR signaling pathway | 38 | 7 |
| Riboflavin metabolism | 38 | 4 |
| Geraniol degradation | 38 | 3 |
| Terpenoid backbone biosynthesis | 37 | 1 |
| Various types of N-glycan biosynthesis | 36 | 8 |
| Caffeine metabolism | 35 | 0 |
| Cyanoamino acid metabolism | 33 | 3 |
| Isoquinoline alkaloid biosynthesis | 33 | 2 |
| Caprolactam degradation | 30 | 4 |
| Steroid biosynthesis | 28 | 0 |
| Butirosin and neomycin biosynthesis | 25 | 3 |
| Synthesis and degradation of ketone bodies | 25 | 1 |
| D-Glutamine and D-glutamate metabolism | 24 | 4 |
| Benzoate degradation | 24 | 2 |
| Styrene degradation | 24 | 2 |
| Sulfur metabolism | 23 | 2 |
| Tropane, piperidine and pyridine alkaloid biosynthesis | 23 | 2 |
| Folate biosynthesis | 23 | 0 |
| Lysine biosynthesis | 22 | 1 |
| D-Arginine and D-ornithine metabolism | 20 | 1 |
| Glycosaminoglycan biosynthesis - heparan sulfate / heparin | 19 | 3 |
| Primary bile acid biosynthesis | 17 | 1 |
| Glycosphingolipid biosynthesis - ganglio series | 16 | 3 |
| Penicillin and cephalosporin biosynthesis | 16 | 3 |
| Steroid hormone biosynthesis | 16 | 3 |
| Fatty acid biosynthesis | 16 | 1 |
| Glycosylphosphatidylinositol(GPI)-anchor biosynthesis | 14 | 1 |
| Phosphonate and phosphinate metabolism | 14 | 1 |
| Toluene degradation | 14 | 1 |
| Ethylbenzene degradation | 14 | 0 |
| Phenylalanine, tyrosine and tryptophan biosynthesis | 14 | 0 |
| Glycosphingolipid biosynthesis - globo series | 12 | 1 |
| Cutin, suberine and wax biosynthesis | 10 | 4 |
| Other types of O-glycan biosynthesis | 10 | 3 |
| Taurine and hypotaurine metabolism | 10 | 3 |
| Ubiquinone and other terpenoid-quinone biosynthesis | 10 | 1 |
| Valine, leucine and isoleucine biosynthesis | 9 | 2 |
| Flavone and flavonol biosynthesis | 8 | 1 |
| Vitamin B6 metabolism | 8 | 1 |
| Diterpenoid biosynthesis | 8 | 0 |
| Photosynthesis | 8 | 0 |
| Glycosaminoglycan biosynthesis - chondroitin sulfate / dermatan sulfate | 7 | 1 |
| C5-Branched dibasic acid metabolism | 7 | 0 |
| Novobiocin biosynthesis | 7 | 0 |
| Biotin metabolism | 6 | 0 |
| Glucosinolate biosynthesis | 4 | 1 |
| Lipoic acid metabolism | 4 | 0 |
| Carbapenem biosynthesis | 3 | 1 |
| Aflatoxin biosynthesis | 3 | 0 |
| Sesquiterpenoid and triterpenoid biosynthesis | 3 | 0 |
| Steroid degradation | 3 | 0 |
| Tetracycline biosynthesis | 3 | 0 |
| Glycosaminoglycan biosynthesis - keratan sulfate | 2 | 1 |
| Betalain biosynthesis | 2 | 0 |
| Biosynthesis of ansamycins | 2 | 0 |
| Indole alkaloid biosynthesis | 2 | 0 |
| Naphthalene degradation | 2 | 0 |
| Peptidoglycan biosynthesis | 2 | 0 |
| PI3K-Akt signaling pathway | 1 | 0 |
| Xylene degradation | 1 | 0 |
